# Primary Percutaneous Coronary Intervention in Single Ostium Coronary Arteries: Challenges and Considerations

**DOI:** 10.7759/cureus.78332

**Published:** 2025-02-01

**Authors:** Muhammad W Saleem, Shafi Ullah, Usman k Zaib, Maha Amjad, Ihsan Ullah, Muhammad Ishaq Khan

**Affiliations:** 1 Cardiology, Peshawar Institute of Cardiology, Peshawar, PAK; 2 Medical School, Rehman Medical College, Peshawar, PAK

**Keywords:** coronary artery anomaly, primary percutaneous coronary intervention (pci), right coronary artery (rca) anomaly, shirani-roberts classification, single ostium coronary artery, st-segment elevation myocardial infarction (stemi)

## Abstract

Coronary anomalies occur in a very small proportion of the general population. One such anomaly is a single ostium coronary artery (SOCA). Primary percutaneous coronary intervention (PCI) in SOCA can pose significant challenges due to anatomical complexity or hardware selection.

We present the case of a 59-year-old man who presented to the emergency department with typical chest pain and electrocardiographic evidence of inferior wall ST-segment elevation myocardial infarction (STEMI). The patient subsequently underwent coronary angiography via a radial approach.

Angiography revealed the left main coronary artery (LMCA) giving rise to a dominant right coronary artery (RCA), anatomically classified as Shirani-Roberts Type 1B. The distal RCA was sub-totally occluded just proximal to the crux. The patient subsequently underwent successful primary PCI to RCA.

Computed tomography coronary angiography (CTCA) is advised in anomalous origin RCA to delineate malignant anatomy. In our case, this was not performed due to the financial constraints of the patient, leaving the exact course of the RCA undetermined. Our case report highlights the importance of operator expertise, catheter selection, and individualized strategies for managing coronary anomalies. It emphasizes that early recognition and tailored procedural planning are critical to optimizing outcomes in patients with SOCA presenting with acute coronary syndromes.

## Introduction

Coronary anomalies have an estimated prevalence of 0.024-0.025% of the general population. Percutaneous coronary intervention (PCI) in such patients is infrequently performed due to technical challenges and high complication rates [1-3].According to a large study by Desmet et al. that reviewed 50,000 consecutive coronary angiograms, only 33 cases of single coronary artery were detected, amounting to a prevalence of 0.066% [4]. A commonly used classification for single ostium coronary arteries (SOCA) is the Shirani-Roberts classification, whereby they are categorized into three major types. In type 1, the osmium arises from the left coronary sinus; in type 2, it arises from the right coronary sinus; and in type 3, it arises from the non-coronary sinus. Each of these types is further subdivided based on the course of the aberrant artery [2]. Due to limited data on the subject, the exact frequencies of each subtype remain unknown. The anatomical classification and clinical frequencies of each subtype hold clinical and surgical significance as certain anatomies can have a malignant course necessitating surgical re-implantation. We present such a case of a SOCA with the right coronary artery (RCA) originating from the left main stem.

## Case presentation

**Figure 1:**
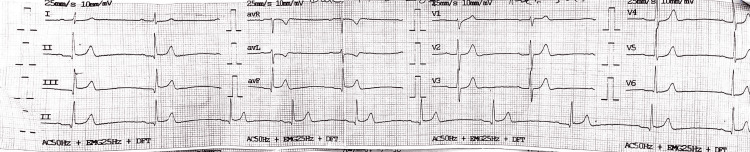
Arrival ECG of the patient. ST-segment elevations are noted in leads II, III, and avF with reciprocal ST-segment depressions in leads I and avL. ECG: electrocardiogram; avF: augmented vector foot; avL: augmented vector left

This data is being reported after obtaining a formal written consent from the patient. The case report is adherent to the guidelines in the Declaration of Helsinki. Personal details of the patient are omitted for confidentiality. The patient is a 59-year-old man who presented to the Accident and Emergency Department (A&E) of Peshawar Institute of Cardiology with a history of typical chest pain of the Canadian Cardiovascular Society (CCS) class 3. The pain started eight hours prior to arrival. His past medical history was insignificant, and he didn't report any risk factors for coronary artery disease. The A&E was his point of first medical contact. On arrival, the patient's vitals were as follows: blood pressure, 132/90 mmHg; pulse rate, 70/min; and oxygen saturation, 94% on ambient air. On examination, he had equal air entry in the chest bilaterally with no added sounds. Cardiovascular examination revealed normal heart sounds with no gallop or murmurs. The rest of the systemic examination was also unremarkable. His electrocardiogram (ECG) showed ST-segment elevation in leads II, III, and augmented vector foot (aVF) with reciprocal ST-segment depressions in leads I and augmented vector left (aVL) (Figure 1). He was diagnosed as a case of inferior wall myocardial infarction (IWMI) and was given a loading dose of aspirin 300 mg, clopidogrel 600 mg, and heparin 5000 IU. He was subsequently shifted to the catheter lab for coronary angiography and primary PCI. 

**Figure 2:**
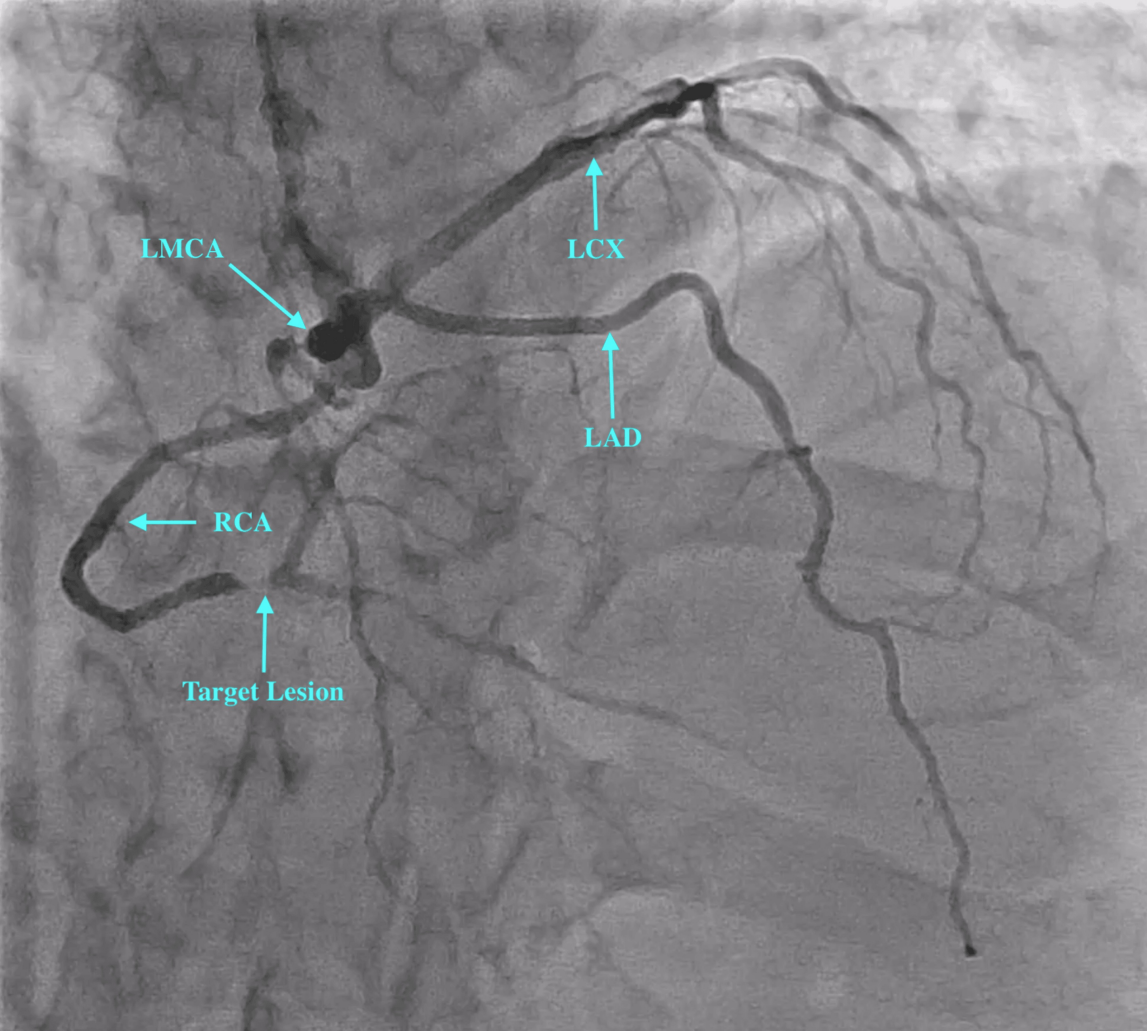
Coronary angiogram prior to PCI in PA cranial view. PCI: percutaneous coronary intervention; PA: posteroanterior; LMCA: left main coronary artery; LAD: left anterior descending artery; LCX: left circumflex artery; RCA: right coronary artery

**Figure 3:**
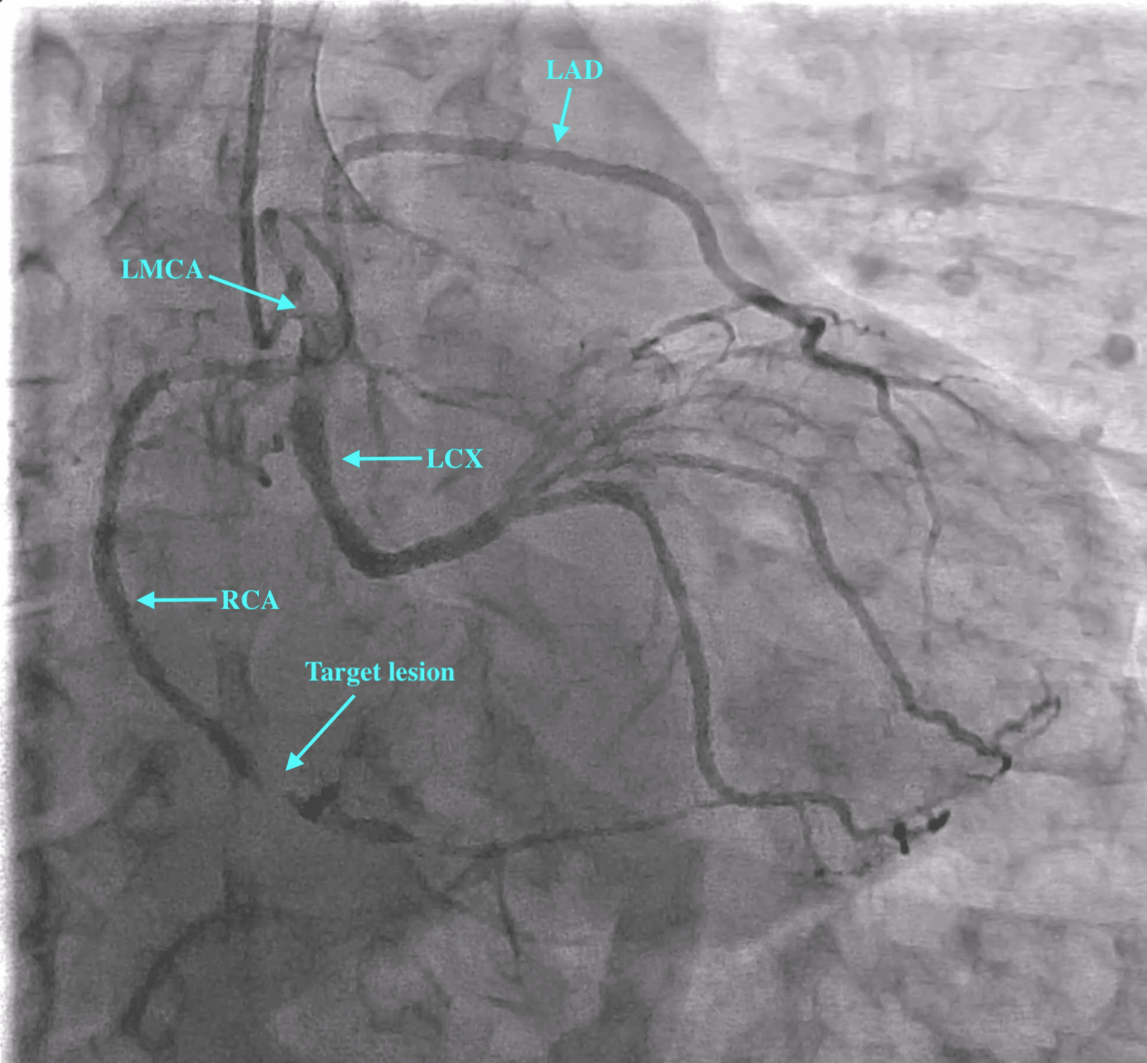
Coronary angiogram prior to PCI in RAO caudal view. PCI: percutaneous coronary intervention; RAO: right anterior oblique; LMCA: left main coronary artery; LAD: left anterior descending artery; LCX: left circumflex artery; RCA: right coronary artery

**Figure 4:**
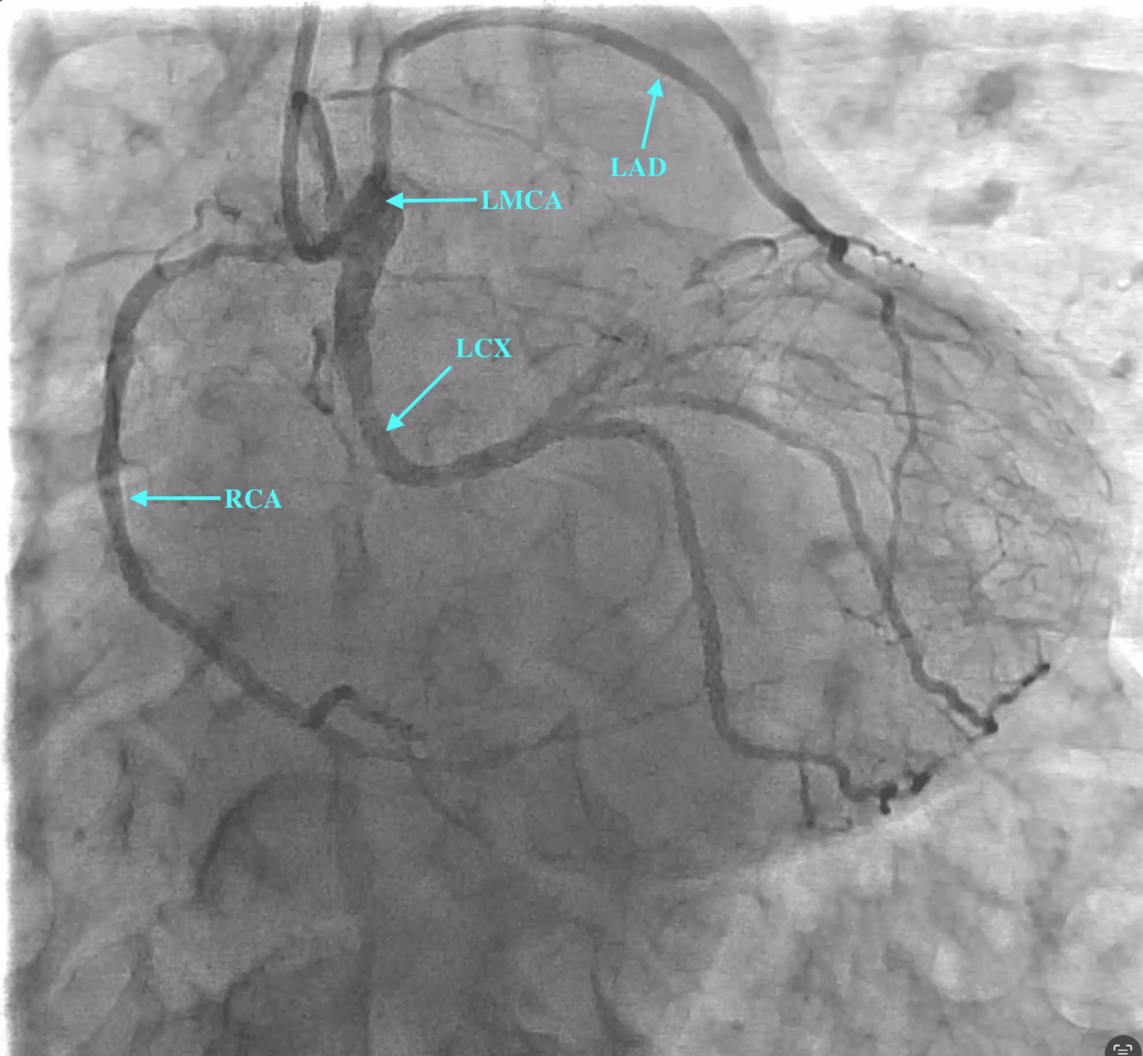
Post-PCI coronary angiogram in PA caudal view. PCI: percutaneous coronary intervention; PA: posteroanterior; LMCA: left main coronary artery; LAD: left anterior descending artery; LCX: left circumflex artery; RCA: right coronary artery

**Figure 5:**
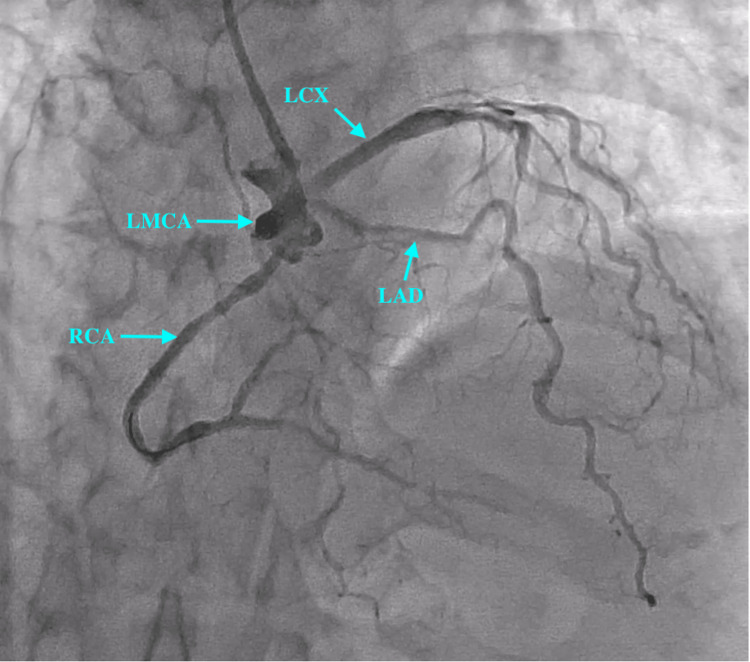
Post-PCI coronary angiogram in PA cranial view. PCI: percutaneous coronary intervention; PA: posteroanterior; LMCA: left main coronary artery; LAD: left anterior descending artery; LCX: left circumflex artery; RCA: right coronary artery

Radial approach was used to gain access with a 6-French radial sheath. A 5-French Tiger catheter was used to engage the coronaries. On engagement of the left coronary ostium, we observed a dominant RCA arising from the left main coronary artery (LMCA) (see Figure 2 and Figure 3). The left circumflex artery (LCX) showed proximal mild stenosis with subsequent severe stenosis of the first obtuse marginal (OM) branch. The RCA showed proximal moderate disease with distal severe disease just proximal to the crux. The left anterior descending artery (LAD) was normal. A decision was made to deploy a stent in the distal RCA. The left coronary ostium was engaged with a 6-French extra-backup (EBU) 3.5 catheter. The lesion was subsequently crossed with a balance middle weight (BMW) wire. The RCA and posterior descending artery (PDA) were stented with a single Promus Premier 2.5×18 mm stent (Boston Scientific, Marlborough, MA, USA) deployed at 12 atmospheric pressure. The stent was then post-dilated with a 2.75×8 mm NC Euphora balloon (Medtronic, Minneapolis, MN, USA). The procedure was successfully completed without any complications, and the Thrombolysis in Myocardial Infarction (TIMI) III flow was established (Figure 4 and Figure 5). The patient was subsequently advised to undergo computed tomography coronary angiography (CTCA) to rule out an anomalous course of the RCA. 

Healthcare services in the province of Khyber Pakhtunkhwa are provided through the National Health Insurance Program known as the Sehat Sahulat Program (SSP). This program encompasses emergency medical procedures and surgical interventions. Regrettably, CTCA is not included in the coverage of this program. Consequently, the patient opted against undergoing CTCA due to financial constraints.

The patient was scheduled for his first follow-up visit four weeks following his initial hospitalization. He reported satisfactory functional status and no symptoms. His medications were adjusted according to the current guideline-directed medical therapies. He was advised to schedule another follow-up appointment in three months.

## Discussion

There are a vast variety of diagnostic and guiding catheters available for use. In a study by Nasrin et al. on 25 consecutive patients with anomalous RCA, the catheters most commonly used to engage the coronary ostia in an anomalous RCA were Judkins left (JL), Judkins right (JR), and multipurpose angled (MPA). In this study, the average catheters used precisely were 2±1. Anchoring wire was used in 12% of cases and guide extension in 8% of cases [5]. Some of the previous studies show that manually manipulated EBU guiding catheter and Ikari right 1.5 catheter were used to cannulate the anomalous RCA arising from the left coronary sinus [6]. In our case, we used an EBU 3.5 catheter without any manipulation and were successful in canulating the LMCA. EBU was used due to operator preference keeping in mind the anatomical origin of the ostium. Furthermore, the selection of a catheter depends on the proper anatomical orientation, size of coronary ostia, and operator preference/familiarity with using a catheter [7]. This was a case of Shirani-Roberts type 1B solitary coronary artery. This type is further subdivided into four subtypes based on the course of the RCA, which requires CTCA for delineation [2]. One of the main concerns with such an anatomy is to rule out anomalous intra-arterial or inter-arterial course of the RCA between the aorta and the pulmonary artery. In case of inter-arterial or intra-arterial course, patients require surgical intervention. The malignant course of coronary arteries can lead to the compression of the coronary artery during systole. Although the coronary flow is mainly diastolic, tachycardia in such patients can reduce diastolic time and cause symptoms. In our case, we were unable to further delineate the anatomy and risk stratify the patient due to financial limitations. Surgical options for the treatment of the anomalous course of RCA include unroofing of the intramural segment, surgical re-implantation, ostial reconstruction, and coronary artery bypass [8,9]. Follow-up interval in such patients should be on a case-by-case basis. The anatomical complexity, lesion characteristics, course of the artery, procedural complications, ejection fraction, and functional status of the patient should all be taken into account. If no significant point of concern is noted, then standard follow-up protocols should be used, which in the case of our institute is 4-6 weeks after primary PCI.

## Conclusions

SOCA can be a shocking finding on initial angiography and can lead to hesitation among operators when PCI is needed. The choice of catheters can be a challenge in such cases, but operator preference, technical expertise, catheter choice, and anatomical knowledge can guide towards successful angioplasty. More research needs to be done regarding the frequencies of the various Shirani-Roberts variants. Follow-up of such patients especially those patients with inter-/intra-arterial course needs to be studied in depth to risk stratify these patients and evaluate current therapeutic approaches.
